# Telomerase insufficiency induced telomere erosion accumulation in successive generations in dyskeratosis congenita family

**DOI:** 10.1002/mgg3.709

**Published:** 2019-05-22

**Authors:** Caixia He, Shuang Jing, Congling Dai, Chaofeng Tu, Zhenhua Tan, Juan Du, Guang‐Xiu Lu, Ge Lin, Sicong Zeng

**Affiliations:** ^1^ Institute of Reproduction and Stem Cell Engineering, School of Basic Medical Science Central South University Changsha China; ^2^ Hunan Guangxiu Hospital Changsha China; ^3^ Reproductive and Genetic Hospital of CITIC‐Xiangya Changsha China; ^4^ School of medicine Hunan Normal University Changsha China

**Keywords:** compound heterozygous mutations, dyskeratosis congenita, telomerase, telomere length, *TERT* gene

## Abstract

**Background:**

Dyskeratosis congenita (DC) is a rare heritable bone marrow failure syndrome that is associated with telomere dysfunction, and has high genetic heterogeneity and varied features.

**Objective:**

This study aimed to identify the underlying genetic etiology of a DC family with more severe symptoms in the younger generation and to explore the relationship between the genetic causes and the severity of DC phenotype.

**Methods:**

Whole‐exome sequencing was performed on the proband to screen the candidate causative gene. The protein structure was then predicted by SWISS‐MODEL software. Telomere length (TL) assay was performed on family members along with large‐scale population controls. The prenatal diagnosis (PND) was performed on the fetus of parents with secondary pregnancy.

**Results:**

Novel heterozygous mutations in *TERT* (NM_198253.2), c.1796G>A (p.Arg599Gln), c.2839T>C (p.Ser947Pro), and c.3346G>C (p.Glu1116Gln) were identified in the proband. His TL was below the first percentile of the peers, which also appeared on the fetus with epidermal dyskeratosis through PND. The TL data of large‐scale population and members of the DC family implied the accumulation of telomere erosion in successive generations in this family.

**Conclusions:**

Our study identified three clinical pathologic *TERT* mutations and implied that telomere erosion might be accumulated through successive generations, contributing to the severity of DC in the younger generation.

## 1 INTRODUCTION

Dyskeratosis congenita (DC) is a rare heritable disorder that is associated with telomere maintenance and marked by short telomeres, subsequently leading to the susceptibility to diseases such as bone marrow failure, cancer, and pulmonary fibrosis. Most of the patients present with a classic triad of oral leukoplakia, reticular skin pigmentation, and nail dystrophy. Though the classic triad may not manifest in all patients, DC patients unusually have short telomeres for their age. Telomeres are nucleoprotein complexes that consist of tandem nucleotide repeats (TTAGGG)n and a protein complex. Telomere length (TL) is largest at birth and is gradually decreased with age (Rizvi, Raza, & Mahdi, 2014). The shortened telomere can be extended by telomerase, which is a ribonucleoprotein complex that consists of telomerase RNA (TREC) and telomere reverse transcriptase (TERT).

The classic triads and other DC‐related physical manifestations often develop at different rates over time, challenging for proper diagnosis (Vulliamy et al., 2006). In DC family, diagnosis can be obscured by subtle physical findings in the earlier generations. Reliable diagnosis of individuals with signs or symptoms that is suggestive of DC required very short telomeres and/or a pathogenic variant of DC‐related genes (such as *TERT* (MIM 187270), *ACD*, *CTC1*, *DKC1*, *NHP2*, *NOP10*, *PARN*, *RTEL1*, *TERC*, *TINF2*, and *WRAP53*) (Savage, 2018). However, DC‐related variations with uncertain clinical significance were often detected by genetic testing. In this situation, analysis of the pathogenicity of these variants is important for us to understand the development of DC disease and also necessary for proper diagnosis, treatment, genetic counseling, and management.

DC is commonly confirmed by molecular diagnosis if a patient's peripheral blood mononuclear cell TL was less than the first percentile of the age‐matched population (Alter et al., 2012) due to decreasing telomerase activity levels (Marrone et al., 2007). Hence, in our study, we identified the genetic causes behind the disease in a family affected by DC. We characterized the pathogenicity of three novel *TERT* mutations by predicting the protein structure, TL measurement, and telomerase assays. Our data further suggested that a continuous dose with insufficient telomerase may affect the clinical penetrance of DC.

## 2 MATERIALS AND METHODS

### 2.1 Ethical compliance

This study was approved by the ethics committee of the Reproductive and Genetic Hospital of CITIC‐Xiangya. Written informed consent was obtained from all participants and legal guardians.

### 2.2 Patient and blood sample

**Figure 1:**
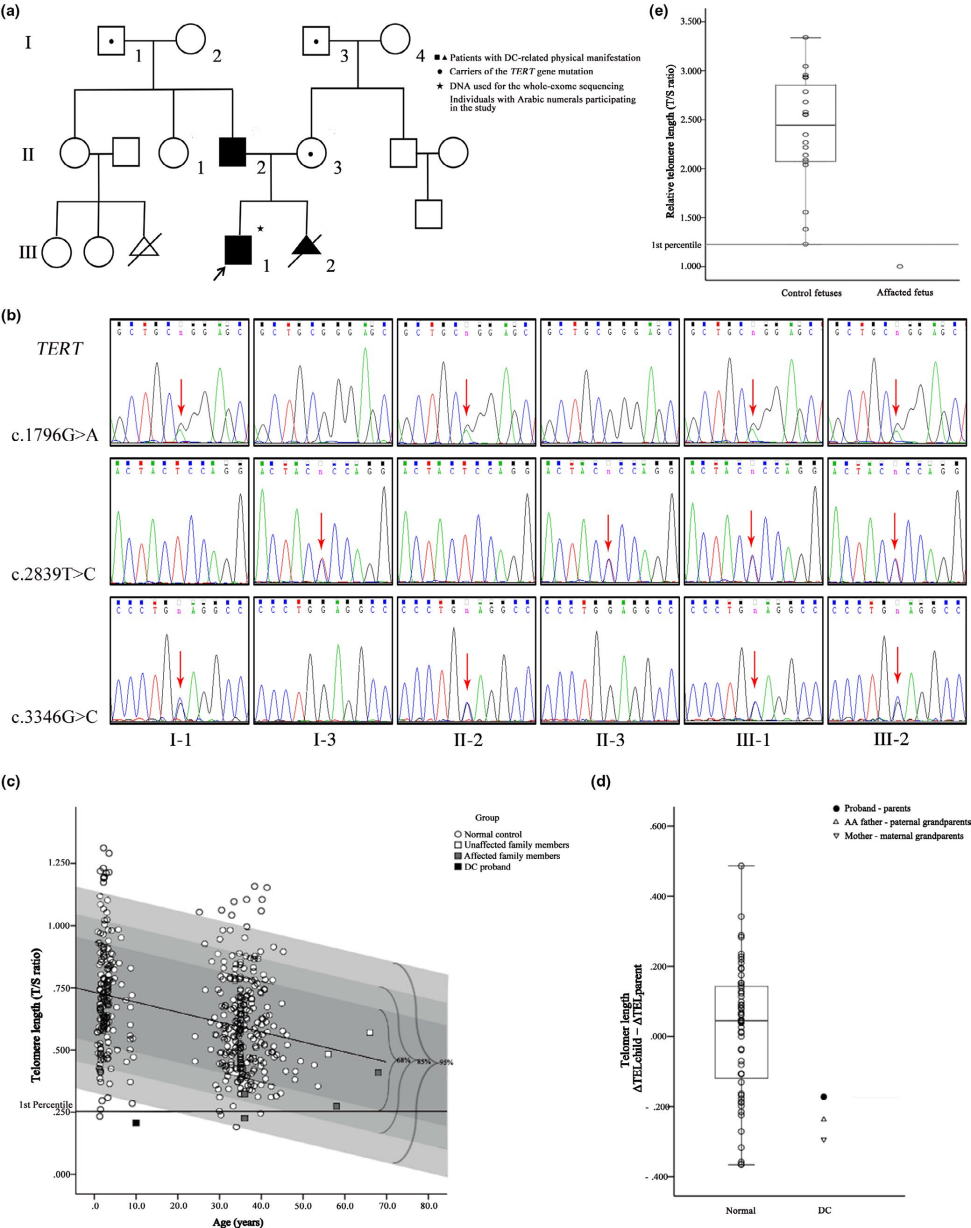
Molecular diagnosis of Dyskeratosis Congenita family. (a) Pedigree of the family. (b) Sequence chromatograms for *TERT* (NM_198253.2, c.1796G>A, c.2839T>C and c.3346G>C) in this family. Red arrow indicates mutation site. (c) Telomere length analysis in the DC family. Telomere length (T/S ratio) in the peripheral blood of eight members of the DC family and the normal controls (435 healthy control subjects between the ages of 0.67 and 53.54 years). The best‐fit‐line through this normal range is presented as a black line that corresponds to the equation Y = 0.758 − 0.004X. Deviation from the best‐fit‐line has been highlighted as a dark gray square of 68%, a lighter gray square of 85%, and the palest gray square of 95%. (T, telomere; S, 36B4 single‐copy gene). (d) Telomere shortening across generations in the DC family. The change in age‐adjusted telomere length measurement between parent‐child combinations (ΔTEL_child_ − ΔTEL_parent_) in 56 normal families and the DC family. Bar of the darkest color indicated median values. (*n* = 56; Mean = 0.015; *p* values were determined by *t* test, *p* < 0.05). (e) The aborted fetal telomere length is far lower than the first percentile of the other 20 miscarried fetuses in similar months. Fetus (gestational age from 12 weeks to 24 weeks) with relative telomere length of skin tissue. (*n* = 20; Mean = 2.385; *P* values were determined by *t* test, *p* < 0.01).

A three generation pedigree with dyskeratosis congenita (Figure 1a) showed three members with DC‐related physical manifestation. Peripheral blood was collected from eight members of the pedigree.

### 2.3 The whole‐exome sequencing and variant filtering

Exome sequencing was performed on proband (III‐1). Sequences were captured through by the Agilent SureSelect version 4 (Agilent Technologies, Santa Clara, CA). The enriched library was sequenced by an Illumina HighSeq2500. The sequencing reads were aligned to the GRCh37.p10 by using BWAv0.59. We then performed local realignment and base quality recalibration of the Burrows–Wheeler aligned reads by using the GATK IndelRealigner and the GATK BaseRecalibrator, respectively. Single‐nucleotide variants and small deletions or insertions were recognized by using the GATK UnifiedGenotyper. The Consensus Coding Sequences Database (20130630) was used to annotate the variants. The obtained variants were filtered and then the variations with minor allele frequencies (MAF) <0.01 were picked.

### 2.4 Sanger sequencing

GenBank sequences of human *TERT* (accession number: NM_198253.2) was referred to at the NCBI Reference Sequence Database. Sanger sequencing was used to verify three mutations of *TERT* gene (c.1796G>A, c.2839T>C, c.3346G>C) using specific primers.

The primers are as follows:

1. *TERT* c.1796 specific primer, forward 5′‐GTCTGTTGTCTGGCTGAGCA‐3′ and reverse 5′‐CCAAATGTGGGGCTCAAACG‐3′;
2. *TERT* c.2839 specific primer, forward 5′‐TTGCGGAAGACAGTGGTGAAC‐3′ and reverse 5′‐GAAGACACCTCAGTGCACCC‐3′;
3. *TERT* c.3346 specific primer, forward 5′‐GGGTGTCTGTCCCTTCACTG‐3′ and reverse 5′‐TCGGCCAAACACTCACTCAG‐3′.

The amplified PCR products were analyzed by 2.0% agarose gel electrophoresis to define the size of the band, and then the bidirectional sequencing was performed by using the ABI 3,730 automated sequencer (Applied Biosystems, Forster City, CA).

### 2.5 In silico protein 3D structure analysis 

The SWISS‐MODEL software (https://swissmodel.expasy.org) was used for structural analysis of the variant.

### 2.6 Telomere length (TL) was measured using real‐time fluorescence quantitative PCR (qPCR)

The *TERT* gene variants were functionally analyzed by real‐time quantitative PCR using DNA by directly extracting from the blood samples of this pedigree and normal control group.

In the meantime, the *TERT* gene variants were functionally analyzed by real‐time quantitative PCR using DNA by directly extracting from the placental tissue. The placental tissue from the experimental and normal control sample was respectively donated by this DC family and a healthy family.

The average TL was analyzed through real‐time quantitative PCR as described previously (Gil & Coetzer, 2004; Zeng, Liu, Sun, Lu, & Lin, 2017) and executed on the Roche LightCycler 480 Platform. Genomic DNA from peripheral blood samples and placental tissue was extracted by using the QiaAmp DNA Mini Kit (Qiagen). The quantity and quality of genomic DNA's were determined through NanoDrop 1000 (Thermo Fisher Scientific). Two PCR runs were conducted for each sample, one for the Ct value of the telomere (T), and the other for the Ct value of the single copy gene (S). Primers for telomere (T) and single copy gene (S) were added to the final concentrations of 400 nM and 300 nM.

The primers (Gil & Coetzer, 2004) are as follows:

tel 1b: 5′‐CGGTTTGTTTGGGTTTGGGTTTGGGTTTGGGTTTGGGTT‐3′;

tel 2b: 5′‐GGCTTGCCTTACCCTTACCCTTACCCTTACCCTTACCCT‐3′;

36B4u: 5′‐CAGCAAGTGGGAAGGTGTAATCC‐3′;

36B4d: 5′‐CCCATTCTATCATCAACGGGTACAA‐3′.

For each PCR reaction, the known amounts of DNA were made through serial dilutions (100, 50, 25, 12.5, 6.3, 3.1, 1.6 ng/reaction) to form a standard curve. The thermal cycling conditions for PCR were as follows: stage 1 for telomere and 36B4 (the enzyme was activated): 95°C for 10 min; and stage 2 for telomere: 35 cycles of 95°C for 5 s, 56°C for 10 s, and 72°C for 60 s with signal acquisition. While stage 2 for 36B4: 40 cycles of 95°C for 5 s, 58°C for 10 s, and 72°C for 40 s with signal acquisition. Melt curve analysis was performed at the end of each run to verify the specificity of PCR amplification products. The LightCycler 480 software 1.5.0 (Roche) was used to generate the standard curves and Ct values of the telomere signals (T) and the single copy gene signals (S). The T/S ratio represents the relative TL. As TL shortens with age, the age‐adjusted value (∆TEL) was obtained by drawing a line of best fit of all control sample measurements plotted against age. A predicted TL value (T/S ratio) for any age was then obtained from this line and was subtracted from the measurement obtained by Q‐PCR for any subject to present a ∆TEL.

### 2.7 Telomerase assays

Quantitative measurement of the telomerase activities of the DC placental tissue (DC‐PT) and the normal placental tissue (CON‐PT) were performed by using the TRAPeze^®^ RT Telomerase Detection Kit (EMD Millipore). This assay quantified the telomerase activity by measuring the real‐time fluorescence emission using qPCR and was performed according to the manufacturer's instructions. Briefly, the placental tissue samples were placed in a sterile mortar and freeze by adding liquid nitrogen. Pulverize the sample by grinding with a matching pestle. Transfer the thawed sample to a sterile 1.5 ml microcentrifuge tube, and resuspend in an appropriate amount of CHAPS lysis buffer on ice for 30 min, followed by centrifugation at 12,000 g at 4°C for 20 min. Telomerase‐positive cells (kit components) and telomerase‐negative samples (heat shock factors; HSFs) were lysed in CHAPS lysis buffer on ice for 30 min, followed by centrifugation at 12,000 g at 4°C for 20 min. The supernatant was collected and the protein concentrations were measured by NanoDrop 1,000. The aliquots of cell lysate (1 μg of protein/well) were assayed in a 96‐well quantitative PCR plate. In addition, the telomerase activity of the telomeric repeats (5′‐GGTTAG‐3′) that was added to the 3′ end of a substrate oligonucleotide (TS) within an initial reaction was then determined by qPCR using the Roche LightCycler 480 Platform. The relative values were computed by comparing the average Ct values of each sample against a standard curve that was generated via using a TSR8 control template. Heat‐inactivated extracts (HSF‐heated) were set as negative controls.

### 2.8 Statistical analysis

Statistical analysis was carried out by using SPSS 23.0 software package. The age‐adjustment and regressions of TL on age were performed by linear regression. The *t* test was used for analyzing the TL results for family members. Statistical significance was defined as *p* < 0.05.

## 3 RESULTS

### 3.1 Clinical features

The proband (III‐1) was a 10‐year‐old boy, with a birth weight of only 2.1 kg. After that, he was diagnosed as having intrauterine growth retardation and cerebellar dysplasia. He could not walk until he was 3 years of age. At age 5, he was presented with repeated subcutaneous hemorrhage in the neck and lower extremities and upper respiratory tract infections, and was diagnosed to have thrombocytopenia. At age 9, he was clinically diagnosed with aplastic anemia (AA). The proband's father (II‐2) was diagnosed with AA at 26‐years, and was recovered after 2 years with cyclosporine and stanozolol treatment. The mother (II‐3) was then 6 months pregnant. (The related clinical symptoms of DC family members were presented in Supplemental Table S1).

### 3.2 Identification of a compound heterozygous variation in *TERT* gene by Whole‐exome sequencing and pedigree analysis

There were 12 variants that met the criterion (Table S2). The site variations in c.1796G>A, c.2839T>C, and c.3346G>C in *TERT* seemed to be the most appropriate causative genetic mutations in DC. Sanger sequencing showed three variation sites in *TERT* in all nine family members (Figure 1b and Figure S1a). In proband (III‐1) and intrauterine fetus (III‐2), two mutations (c.1796G>A and c.3346G>C) in *TERT* gene were inherited from the father (II‐2) with a history of AA, and the paternal grandfather (I‐1) also had the same variants. While heterozygous variant c.2839T>C (p.Ser947Pro) in *TERT* gene was inherited from the mother (II‐3) with the earlier onset of gray hair and the maternal grandfather (I‐3) also had the same variant.

### 3.3 SWISS‐MODEL software revealed the three key variation sites of *TERT*

**Figure 2:**
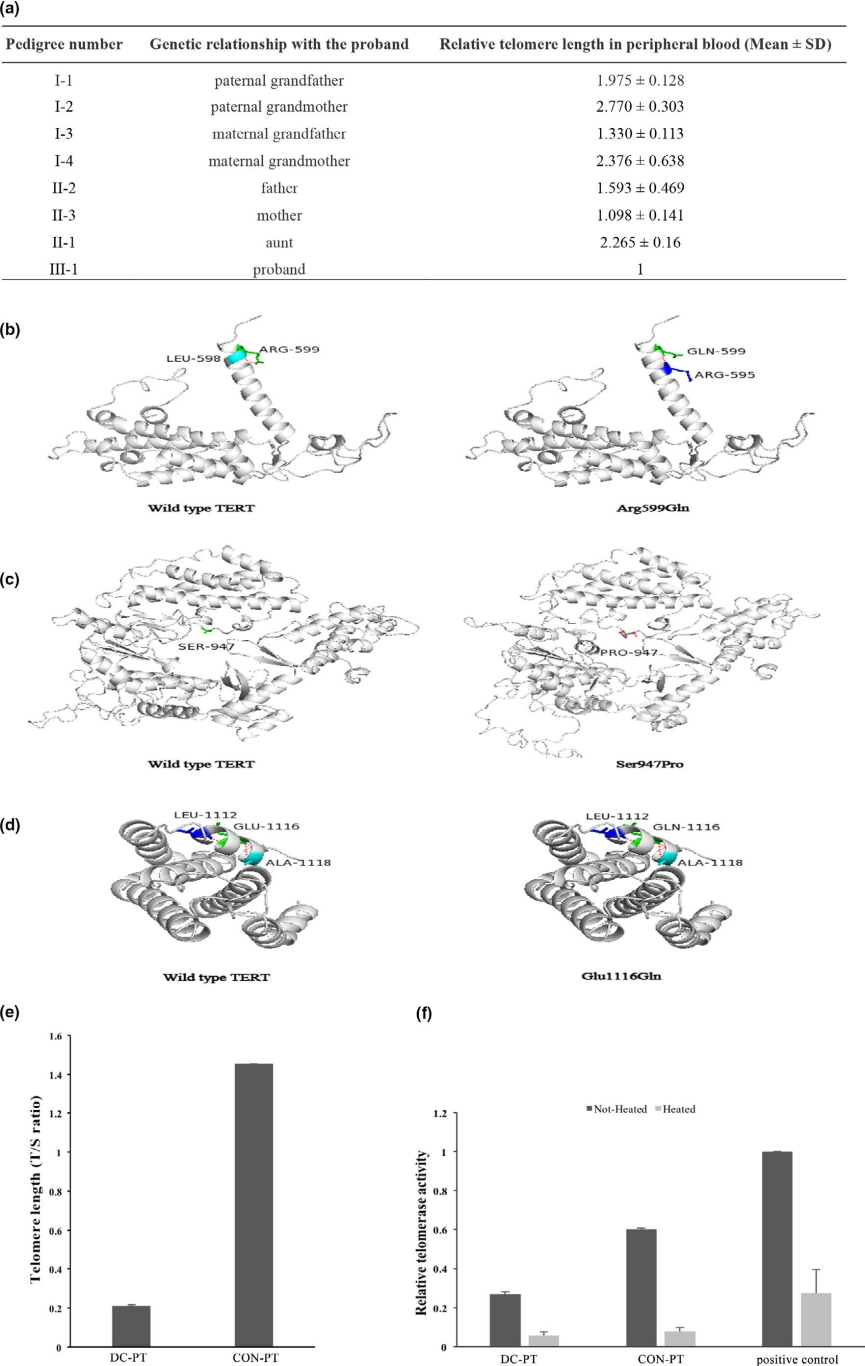
The function of *TERT* mutations. (a) Relative telomere length of the DC family members (Mean ± *SD*, *n* = 3). (b–d) The modeling of three mutation sites of c.1796G>A (p.Arg599Gln), c.2839T>C (p.Ser947Pro), and c.3346G>C (p.Glu1116Gln) in *TERT* gene (NM_198253.2). (e) Telomere length analysis in the placental tissue (PT). Telomere length (T/S ratio) for DC‐PT was lower than that of CON‐PT (placental tissue of the dyskeratosis congenita fetus: DC‐PT and the healthy fetus: CON‐PT). (f) Relative telomerase activity of DC‐PT and CON‐PT

TERT is composed of three domains, the N‐terminal extension (NTE) that contained the RNA‐interaction domains 1 and 2 (RID1 and RID2), reverse transcription domain (RT) where the nucleotide transfer occurs, and a C‐terminal extension (CTE) for processivity and localization. The p.Arg599Gln amino acid substitution is positioned near the RNA‐interaction domains, while the p.Ser947Pro and p.Glu1116Gln amino acid substitutions are both located in the CTE domain. The SWISS‐MODEL software revealed that the three missense variations in *TERT* gene caused altered protein structure (Figure 2b–d). Hydrogen bond between Arg599 and Leu598 was ruptured, while Arg was replaced by Gln in *TERT*599, forming another hydrogen bond between Gln599 and Arg595 (Figure 2b). Ser was substituted by Pro in *TERT*947, resulting in the replacement of a polar uncharged residue (S) with a nonpolar residue (P) at position 947 of the telomerase protein (Figure 2c). *TERT* c.3346G>C (p.Glu1116Gln) resulted in the replacement of a polar charged and acidic residue (E) with a polar uncharged residue (Q) at position 1,116 of the telomerase protein (Figure 2d).

### 3.4 Telomere length (T/S ratio) in peripheral blood

TL less than the first percentile for age in lymphocytes was 97% sensitive and 91% specific for DC (Alter et al., 2012). In individuals with complex or atypical DC, the test for the lymphocytes could help in the clinical diagnosis. To evaluate the TL of this family, we obtained the normal age‐dependent TL distribution in peripheral blood mononuclear cells (PBMCs) from 435 healthy control subjects between the ages of 0.67 and 53.54 years. As the TL was shortened with age, the age‐adjustment and regressions of TL on age were performed by linear regression. A line of best fit (the equation Y = 0.758 − 0.004X) was drawn through all control sample measurements plotted against age (Figure 1c) (Marrone et al., 2007). With this, we calculated the age‐adjusted value of TL, called ΔTEL (the difference between the actual value and the predicted value), for each individual (Brummendorf, Maciejewski, Mak, Young, & Lansdorp, 2001).

The investigation of TL (Figure1c) showed that the TL of the proband was lower than the first percentile of the peers, supporting the diagnosis of DC. The TL of the proband was presented outside the 95% deviation area of the normal range (black small square in Figure 1c). The heterozygous mother was located within 95% deviation area of the normal range (the palest gray square in Figure 1c). The TL of the father and the maternal grandfather (I‐3) were located within the 85% deviation area (lighter gray square in Figure 1c). The paternal grandfather (I‐1) and the three unaffected members of the family were located within the 68% deviation area (dark gray square Figure 1c). The ΔTEL_child_ − ΔTEL_parent _(T. Vulliamy et al., 2004) of the DC family was a negative value and was below than the normal families (Figure 1d). Furthermore, we presumed that the effect of insufficiency of telomerase reverse transcriptase in this family might accumulate the telomere erosion in successive generations. Then, we evaluated the TL directly by Q‐PCR and observed a pattern of progressive telomere shortening with generations in this DC family (Figure 2a).

### 3.5 Prenatal diagnosis (PND) of affected fetus

The parents decided to terminate this pregnancy (III‐2) as the fetus carried the same *TERT* compound heterozygous mutations as the proband through prenatal diagnosis. The TL of the fetal skin tissue was far below the first percentile (Figure 1e) when compared with other miscarried fetuses in a similar month. TL (T/S ratio) of DC‐PT (placental tissue of the dyskeratosis congenita fetus) was lower than that of CON‐PT (placental tissue of the healthy fetus), (Figure 2e). The telomerase activity assay of the placental tissue showed that the telomerase activity of DC‐PT was lower than that of CON‐PT (Figure 2f). Moreover, pathological examination of the fetus showed epidermal dyskeratosis, folliculitis (H&E staining showed in Figure S1b); increased cardiac empty space; gastric cavity dilatation, mucosal thinning; cerebral ependymal cell hyperplasia and umbilical cord edema.

## 4 DISCUSSION

The classic triad of oral leukoplakia, reticular skin pigmentation, and nail dystrophy is considered extremely important DC clinical features. In this DC family, others had no obvious triad, except for the fetus who has epidermal dyskeratosis. Meanwhile, the fetus had pathogenic variants of *TERT*, short telomeres, and low telomerase activity. The proband was considered with a specific and severe DC‐Hoyeraal Hreidarsson syndrome based on very short telomeres and pathogenic variants of *TERT* and clinical characterization of it included intrauterine growth retardation, cerebellar dysplasia, motor retardation, and AA. According to the previous reports, some of the DC cases carried mutation in the *TERT* gene, showing the DC classic triads (Marrone et al., 2007), while few other did not (Basel‐Vanagaite et al., 2008). The DC family indicated that the appearance of triad symptoms had individual differences among the DC patients, even in the same family with same *TERT* genotype.

Clinically, the maternal grandfather had no obvious clinical symptoms, while the paternal grandfather had low white cell count and hemoglobin which were not enough to be diagnosed with anemia. The father had AA and the mother had early onset of gray hair. Severe form of DC was observed in their offspring. The clinical symptoms seemed to be very early onset and more severe for the younger generation. Meanwhile, the TL was continuously shortened in successive generations. A similar situation occurred in the telomerase‐deficient mice, while the first generation (G1) *mTR^−/−^* mice showed no distinct phenotype. Nevertheless, further crossbreeding showed a series of phenotypes, worsening with the continuation of telomere shortening (Hao et al., 2005). Early mammalian embryos experienced telomerase‐dependent TL reset (Ozturk, Sozen, & Demir, 2014; Schaetzlein et al., 2004) to elongate the telomeres to a defined length, possibly requiring to ensure adequate telomere reserves for species integrity (Schaetzlein et al., 2004). We speculated that the telomerase‐dependent genetic program could not be performed normally due to telomerase deficiency during early embryonic development in DC patients. Consequently, during the early embryonic development stage, the offspring's who had telomerase deficiency could not extend the telomere to a specific length by telomerase and could inherit the parental shortened TL. Telomere erosion accumulation in successive generations may be responsible for the early onset and more serious phenotype in DC patient, which was observed in many DC families (Armanios et al., 2005; T. Vulliamy et al., 2004) and in our case.

After receiving genetic counseling, the couple decided to adopt preimplantation genetic diagnosis (PGD) to give birth to healthy children. Due to complex relationship between the genotype and the phenotype of DC disease and the reduction in TL in successive generations of this DC family, embryos carrying only *TERT* mutations from either of the parents may have a certain risk for developing the disease. However, it is still unclear if the embryos from the normal *TERT* genotype of *TERT ^+/−^* parents with short telomeres and occult diseases can recover the shortened telomeres to normal length. Therefore, the progeny of normal *TERT* genotypes with *TERT ^+/−^* parents who have short telomeres and occult diseases is highly desirable for TL measurements, reasonable physical examinations and health monitoring.

## CONFLICT OF INTEREST

The authors declare no conflict of interest.
