# Supplementary material for: Telomerase insufficiency induced telomere erosion accumulation in successive generations in dyskeratosis congenita family
Source: Mol Genet Genomic Med. 2019 May 22;7(7):e00709. doi: 10.1002/mgg3.709 (PMC6625126; doi:10.1002/mgg3.709)

**Supplemental Figure 1.** (A) Sequence chromatograms for *TERT* (NM_198253.2, c.1796G>A, c.2839T>C and c.3346G>C) of unaffected three members in the DC family and placental tissue (DC-PT and CON-PT). (B) The fetus epidermal immunohistochemistry (H&E staining) showed: epidermal dyskeratosis (black arrow indication).


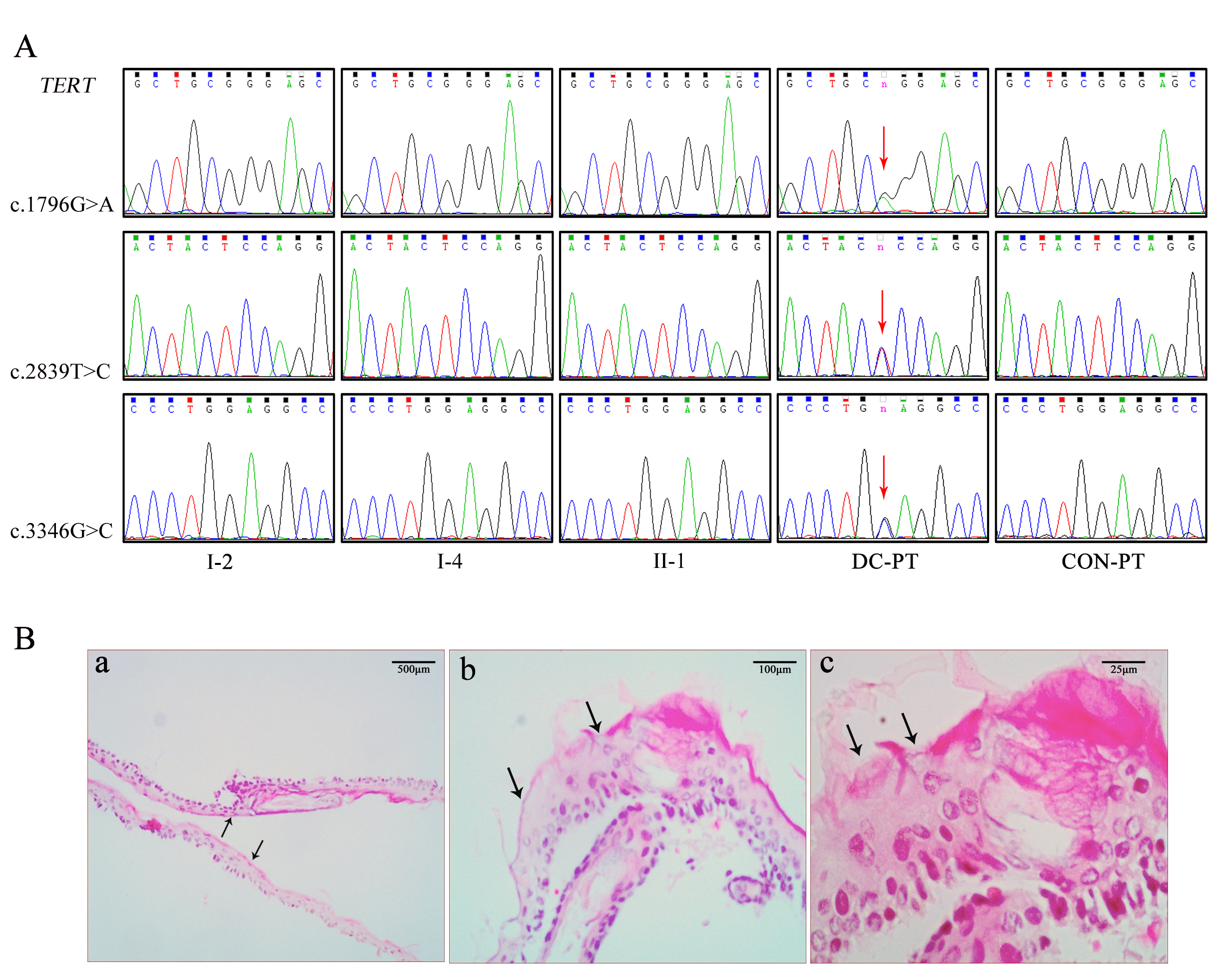

Supplement: Supplementary file 1 [file MGG3-7-e00709-s001.docx]
