# Supplementary material for: Telomerase insufficiency induced telomere erosion accumulation in successive generations in dyskeratosis congenita family
Source: Mol Genet Genomic Med. 2019 May 22;7(7):e00709. doi: 10.1002/mgg3.709 (PMC6625126; doi:10.1002/mgg3.709)
Supplement: Supplementary file 2 [file MGG3-7-e00709-s002.docx]

**Supplemental Table 1. Related clinical symptoms in the DC family members**


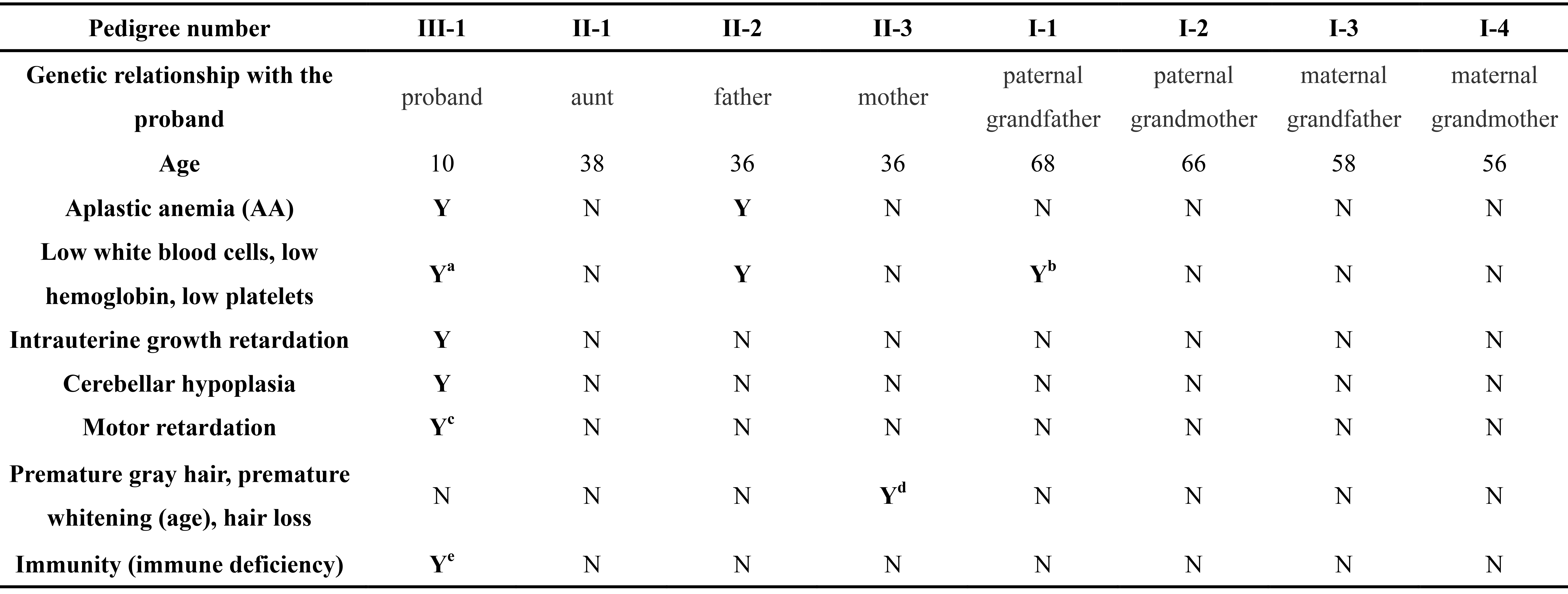


N, no (negative); Y, yes (positive). Other clinical symptoms, ‘Hyperkeratosis of palms and soles, Dysplastic nails, Chest and / or neck mesh pigmentation, Oral leukoplakia, Progressive bone marrow failure (BMF), Myelodysplastic syndrome (MDS), Acute myeloid leukemia (AML), Osteoporosis, Microcephaly, Intracranial calcification, Growth retardation, Height and weight abnormality, Pulmonary Fibrosis, Liver Fibrosis, Solid tumor (head and neck squamous cell carcinoma or anal cancer), Dental caries, abnormal teeth (congenital missing teeth, abnormal growth), periodontal disease, Expanded pulp cavity or reduced root/crown ratio, Tears (excessive tears), Eyelid inflammation, Abnormal eyelashes’ were not found in the DC family at present.

**^a^**: He was diagnosed as to have thrombocytopenia at age 5 (platelets of 8×10^9^/L, white blood cells 3.07×10^9^/L, red blood cells 2. 72×10^12^/L, hemoglobin of 93 g/L and absolute neutrophils count 1.02×10^9^/L).

**^b^**: He had low white cell count and hemoglobin.

**^c^**: The movements of running and jumping are worse, and the balance is poor.

**^d^**: When she was 16 years old, she had white hair. Now, compared with her peers, more white hair is concentrated on her forehead.

**^e^**: He had poor immunity and was prone to infection.
