# Supplementary material for: Telomerase insufficiency induced telomere erosion accumulation in successive generations in dyskeratosis congenita family
Source: Mol Genet Genomic Med. 2019 May 22;7(7):e00709. doi: 10.1002/mgg3.709 (PMC6625126; doi:10.1002/mgg3.709)

**Supplemental Table 2. The list of 12 variants (minor allele frequencies <0.01) associated with blood disorders and presented in patient**


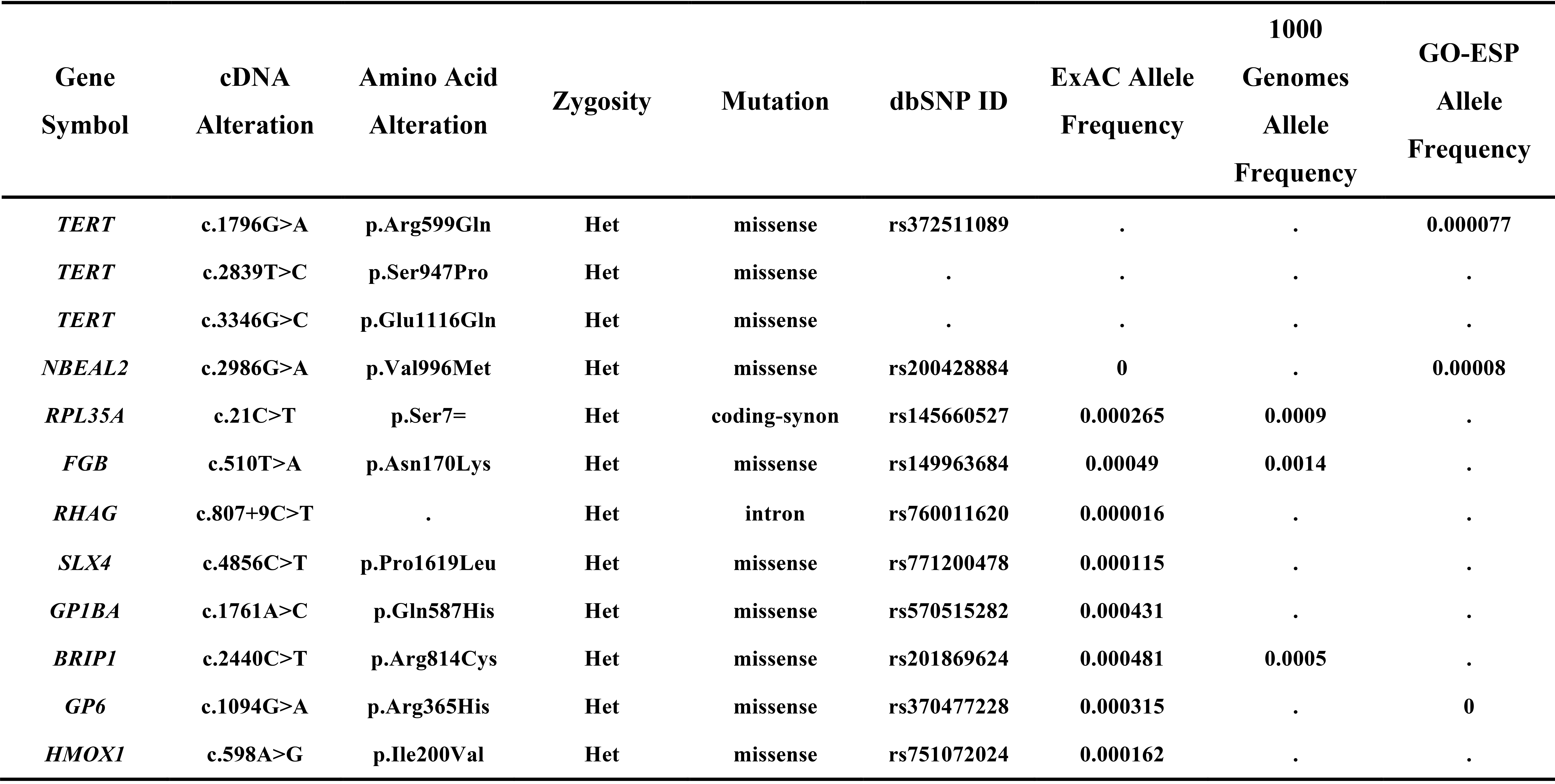

Supplement: Supplementary file 3 [file MGG3-7-e00709-s003.docx]
